# Genomic analysis and clinical implications of immune cell infiltration in gastric cancer

**DOI:** 10.1042/BSR20193308

**Published:** 2020-05-20

**Authors:** Ming Wu, Yadong Wang, Hang Liu, Jukun Song, Jie Ding

**Affiliations:** 1 Medical College, Guizhou University, Guiyang 550025, Guizhou, China; 2 Department of Medicine Emergency, Guizhou Provincial People’s Hospital, Guiyang 550002, Guizhou, China; 3 Department of Oral and Maxillofacial Surgery, Guizhou Provincial People’s Hospital, Guiyang 550002, Guizhou, China; 4 Graduate School, Zunyi Medical University, Zunyi 563003, Guizhou, China; 5 Department of Gastrointestinal Surgery, Guizhou Provincial People’s Hospital, Guiyang 550002, Guizhou, China

**Keywords:** CIBERSOFT, gastric cancer, genomic analysis, immune cell infiltration

## Abstract

The immune infiltration of patients with gastric cancer (GC) is closely associated with clinical prognosis. However, previous studies failed to explain the different subsets of immune cells involved in immune responses and diverse functions. The present study aimed to uncover the differences in immunophenotypes in a tumor microenvironment (TME) between adjacent and tumor tissues and to explore their therapeutic targets. In our study, the relative proportion of immune cells in 229 GC tumor samples and 22 paired matched tissues was evaluated with a Cell type Identification By Estimating Relative Subsets Of known RNA Transcripts (CIBERSORT) algorithm. The correlation between immune cell infiltration and clinical information was analyzed. The proportion of 22 immune cell subsets was assessed to determine the correlation between each immune cell type and clinical features. Three molecular subtypes were identified with ‘CancerSubtypes’ R-package. Functional enrichment was analyzed in each subtype. The profiles of immune infiltration in the GC cohort from The Cancer Genome Atlas (TCGA) varied significantly between the 22 paired tissues. TNM stage was associated with M1 macrophages and eosinophils. Follicular helper T cells were activated at the late stage. Monocytes were associated with radiation therapy. Three clustering processes were obtained via the ‘CancerSubtypes’ R-package. Each cancer subtype had a specific molecular classification and subtype-specific characterization. These findings showed that the CIBERSOFT algorithm could be used to detect differences in the composition of immune-infiltrating cells in GC samples, and these differences might be an important driver of GC progression and treatment response.

## Introduction

Gastric tumor remains one of the most common and heterogeneous neoplasms in the world. Neoplasms pose an important diagnostic and therapeutic challenge in contemporary clinical gastroenterology. They also remain among the top ten major cancer diseases worldwide [1,2]. In China, gastric cancer (GC) is among the top three causes of incidence rates and mortality [3], and the 5-year survival rate of GC is low [1,4]. GC is treated with surgery, radiochemotherapy, immunotherapy and targeted approaches with anti-angiogenic monoclonal antibodies and tyrosine kinase inhibitors, if tumors harbor a specific mutation. Although surgery, chemotherapy, radiotherapy and molecularly targeted drug therapy have provided a means for GC treatment, the prognosis of patients with GC is still not optimistic, and the 5-year overall survival (OS) rate remains low. Biotherapy has achieved certain effects on GC defined by immune cells recruited to and activated in a tumor microenvironment (TME) [5], but the role of immune cells in a TME remains poorly understood.

Tumor inflammatory response has played an important role in cancer occurrence and progression. Several studies have shown that tumor-infiltrating immune cells (TIICs) can help hosts resist cancer cells and promote solid tumor development [6]. As immuno-sensitive tumors, numerous TIICs including T and B lymphocytes and neutrophils are present in the tumor stroma. Cell density and type are closely related to the clinical outcomes of tumors [7–9]. In previous studies, TIICs have been examined by immunohistochemical methods which rely on a single marker to identify a specific TIIC subgroup [10–12]. As such, these approaches are not comprehensive and can be misleading. When few cells are detected or closely related to the cell type, immunohistochemical effect is poor. Consequently, few studies have elucidated the prognostic value of these TIIC subgroups.

Cell type Identification By Estimating Relative Subsets Of known RNA Transcripts (CIBERSORT) [13] has been developed as a new system biology tool which can identify 22 types of immune cells based on transcriptome data. In this research, the gene expression data of 407 patients were collected from The Cancer Genome Atlas (TCGA), and 22 TIIC subsets of GC immune cells were quantified using CIBERSORT for the first time. The relationship between immune cells and clinical features was also explored. Our findings revealed the immunogenomic phenotypes of GC subclasses and provided novel insights into GC immunotherapy and the relationship of complex GC progression, tumor molecular subtypes and immune cell heterogeneity.

## Methods

### Gene expression datasets

The GC dataset including basic information, gene expression profiles and corresponding prognosis information was downloaded from the publicly available dataset of TCGA [14]. Only patients with confirmed GC and clinicopathological and survival information were included in the study. Patients with insufficient or missing datasets such as age, TNM stage and OS, were excluded in the subsequent treatment. RNA sequencing data were converted using the voom (variance modeling at the observational level) method [15,16] and counted data were transformed to values closer to microarray results.

### TIIC quantification via the CIBERSOFT algorithm

The CIBERSORT algorithm was used to determine the immune-related signature from 547 marker immune genes and quantify the relative fraction of each immune cell type. The method was employed to infer their relative proportions of 22 immune cells. Gene expression datasets were written by using standard annotation files, and data were uploaded to the CIBERSORT web and run by using the default signature matrix with 1000 kinds of arrangement. With the CIBERSORT algorithm, *P*-values were obtained for each deconvolution sample via Monte Carlo sampling, which could make each result credible.

The total number of T cells was estimated as the sum of the fraction of CD8^+^ T cells, CD4^+^ naïve T cells, CD4^+^ memory resting T cells, activated CD4^+^ memory T cells, follicular helper T cells, regulatory T cells (Tregs) and γδ T cells. The total macrophage fraction was calculated as the sum of the fractions of M0, M1 and M2 macrophages. The total number of B cells was determined as the sum of memory and naïve B cells.

### Survival analysis

Twenty-two human immune cell phenotypes were further screened. Univariate Cox analysis and Kaplan–Meier survival analysis were performed on LM22 and overall survival (OS) by using the ‘survfit’ function of the survival package in R software. The relationship between clinical feature (TNM stage, radiotherapy, grade) and LM22 was also explored.

### Identification of molecular subtypes

A consensus clustering algorithm was applied to determine the number of clusters and further explore different patterns of TIICs by using the ‘CancerSubtypes’ R-package [17]. Differentially expressed genes (DEGs) and different immune cell types were determined with the Limma R-package to explore the differences in TIICs among each cluster. When DEGs in the dataset were expressed with |log2 fold-change| ≥ 0.1 and adjusted *P*<0.05 was set as the selection standard for subsequent analysis.

### Functional and pathway enrichment analysis

Gene Ontology (GO) [18] and Kyoto Encyclopedia of Genes and Genomes (KEGG) (http://www.genome.ad.jp/kegg/) [19] are the most commonly used tools to describe molecular biology information, such as gene functions, biological functions, protein networks and genomic information. ‘ClusterProfiler’ R package was used to conduct functional and pathway enrichment analysis and reveal the potential biological significance of DEGs among each subtype with a cut-off point of |log2 fold-change| ≥ 0.2 and *P*<0.05. Then, gene set variation analysis (GSVA) revealed the overall pathway of the gene set pathway scores of each sample. Gene sets with c2-curated signatures were downloaded from the Molecular Signature Database of Broad Institute. The obvious enrichment pathway was determined with a threshold of |log fold-change| ≥ 0.2 and adjusted *P*<0.05.

### Statistical analyses

Samples with *P*<0.05 calculated with CIBERSORT were included in the analysis. After they were initially screened, eligible samples were divided into two groups: paired tumor group and paired adjacent tissue group. Pearson correlation analysis was performed with the R software to explore the mutual relationship among three clusters. A Wilcoxon test was conducted to examine the statistical significance between the two groups. A Kruskal–Wallis test was performed to compare the differences between the two groups. A log-rank test was carried out and Kaplan–Meier curves were obtained to examine the differences in OS between the groups.

In the univariate Cox regression analysis, variables with *P*<0.05 were considered as independent prognostic factors, and prognostic LM22 immune cells were further analyzed through multivariate COX regression analysis. The area under the curve (AUC) and the confidence interval under ROC curves were calculated using the R package ‘pROC’ which was used to evaluate the diagnostic accuracy of prognostic LM22 immune cells. Statistical analysis was conducted using software packages provided by the R-Language (R-project.org) and Bioconductor project (www.bioconductor.org).

All analyses were performed using R version 3.3.0. All *P*-values were two-sided, and any *P*<0.05 was considered statistically significant.

## Results

### Overview of data

**Figure 1:**
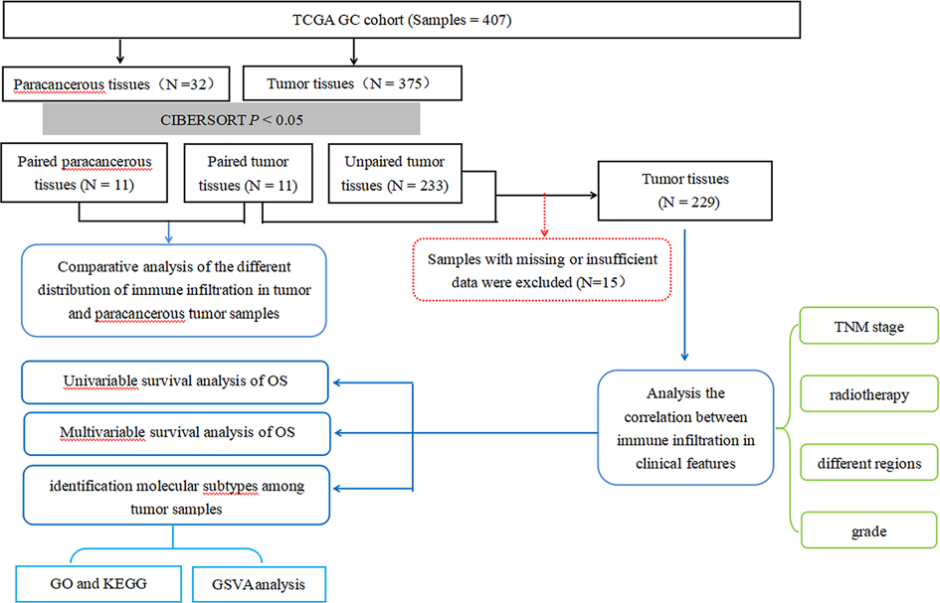
Flowchart detailing the study design and samples at each stage of analysis

The flowchart of study design and the analysis process is exhibited in Figure 1. A total of 407 samples including 32 adjacent-tumor samples and 375 tumor samples were obtained from the TCGA. After the CIBERSOFT algorithm was used, the sample with *P*<0.05 including 15 paracancerous tissues and 244 tumor tissues were considered in the main research contents. After the samples were matched, 22 cases of paired adjacent tumor tissues and paired tumor tissues were selected. The expression profiles of 547 TME-related genes were extracted for further analysis.

### Overview of immune infiltration in GC

**Figure 2:**
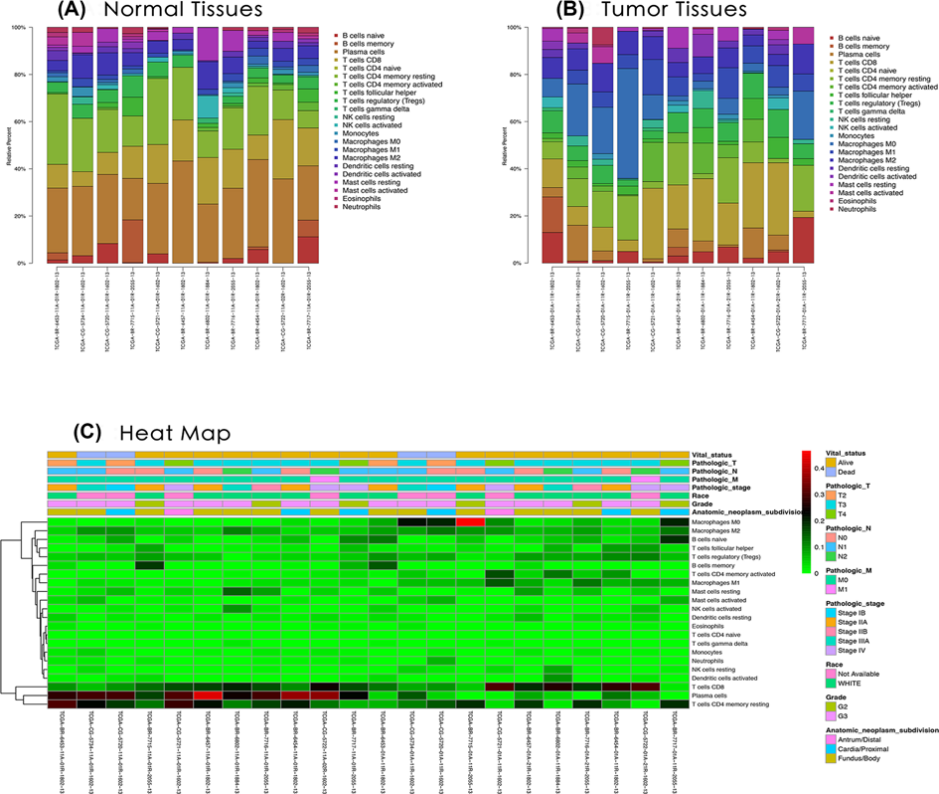
The performance of CIBERSOST for estimating TIICs composition in GC (**A**) Relative proportions of 22 TIICs subpopulation in normal samples. (**B**) Relative proportions of 22 TIICs subpopulation in tumor samples. (**C**) Heatmap of the 22 immune cells’ proportions.

**Figure 3:**
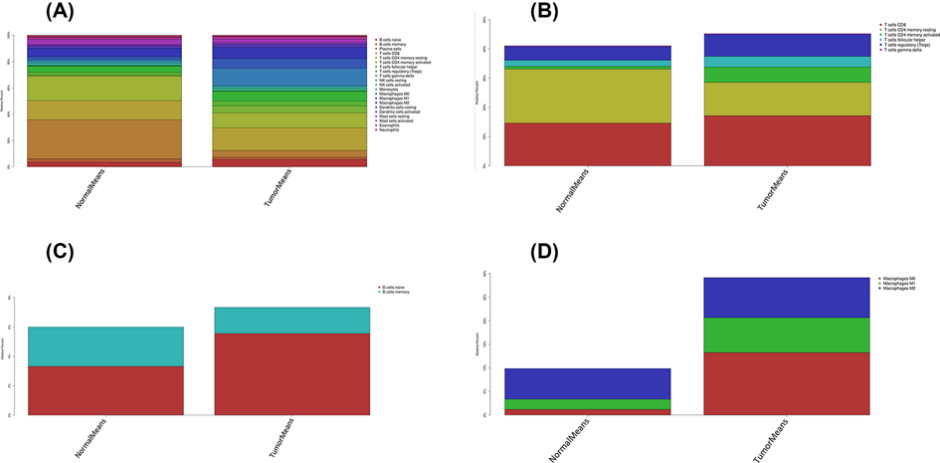
The distribution of 22 immune cell infilteration between GC adjacent tissues and tumor tissues The stacked histogram shows the distribution of 22 immune cell infiltration between GC adjacent tissues and tumor tissues, including total immune cells (**A**), total T cells (**B**), total B cells (**C**) and total macrophages (**D**).

**Figure 4:**
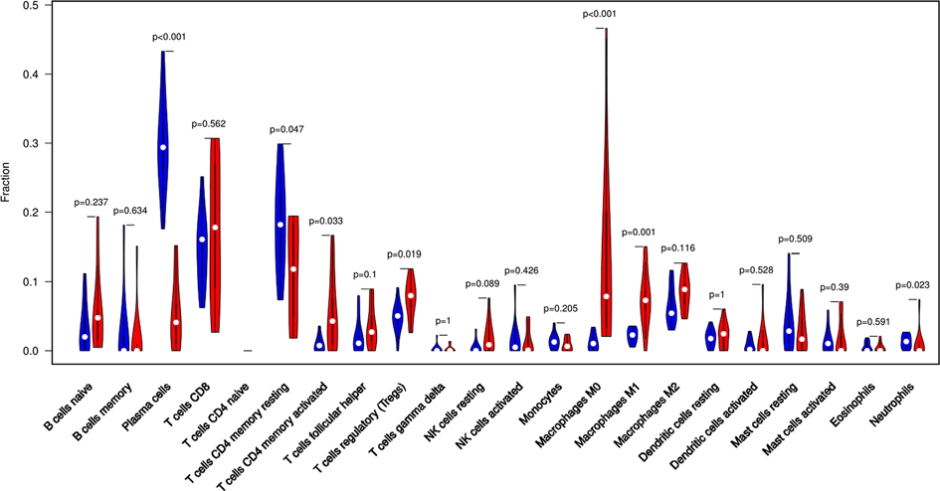
The violin plot exhibits the difference between CIBERSORT immune cell fractions between GC adjacent tissues and tumor tissues

The difference between paired cancer and adjacent tissues in 22 immune cells was analyzed by using the CIBERSORT algorithm. Figure 2 summarizes the results obtained from 22 pairs of the matched patients. Obviously, the proportion of immune cells in GC tumor tissues was significantly different from that in normal tissues. Therefore, changes in TIICs proportions might be intrinsic to individual differences. In Figure 3, the fractions of total T cells, total macrophages and total B cells were higher in GC tumor tissues than in adjacent tumor tissues. Plasma cells, resting CD4 memory T cells, activated CD4 memory T cells, M0 macrophages and M1 macrophages significantly changed between adjacent tumor and tumor groups. The fractions of activated CD4 memory T cells, Tregs, M0 macrophages, M1 macrophages and neutrophils were higher in the tumor samples than in the adjacent tumor samples. The fractions of plasma cells and resting CD4 memory T cells were higher in the adjacent samples than in the tumor samples (Figure 4, Table 1).

**Table 1:** Comparison of CIBERSORT immune cell fractions between normal tissues and tumor tissues in GC

Immune cell type	CIBERSORT fraction in % of all infiltrating immune cells			
	*P*-value	Normal means	Tumor means	logFC
Naive B cells	0.237	0.033	0.055	0.742
Memory B cells	0.634	0.027	0.018	−0.590
Plasma cells	<0.001	0.297	0.052	−2.523
CD8 T cells	0.562	0.146	0.172	0.230
Naive CD4 T cells	NA	NA	NA	NA
Resting CD4 memory T cells	0.047	0.185	0.114	−0.700
Activated CD4 memory T cells	0.033	0.009	0.053	2.484
Follicular helper T cells	0.100	0.021	0.036	0.814
Tregs	0.019	0.047	0.075	0.674
γδ T cells	1	0.003	0.002	−0.153
Resting NK cells	0.089	0.005	0.020	1.896
Activated NK cells	0.426	0.016	0.012	−0.393
Monocytes	0.205	0.014	0.008	−0.769
M0 macrophages	<0.001	0.012	0.133	3.472
M1 macrophages	0.001	0.022	0.074	1.769
M2 macrophages	0.116	0.065	0.085	0.387
Resting dendritic cells	1	0.019	0.022	0.200
Activated dendritic cells	0.528	0.008	0.014	0.793
Resting mast cells	0.509	0.040	0.026	−0.596
Activated mast cells	0.390	0.013	0.017	0.327
Eosinophils	0.591	0.005	0.004	−0.308

NA: not available.

The proportions of different TIICs were moderately to strongly correlated in the GC-paired adjacent tumor samples. The mutual relationship in LM22 immune cells reduced in the tumor samples. For examples, follicular helper T cells were highly and positively associated with memory B cells in the immune phenotype profiles in the TMC from the GC-paired adjacent tumor samples, whereas CD8 T cells were highly and negatively related to resting CD4 memory T cells in the GC-paired adjacent tumor group (Supplementary Figure S1). Therefore, we hypothesized that alterations in the TME cell infiltration rate directly reflected differences in immunity between the two groups.

**Figure 5:**
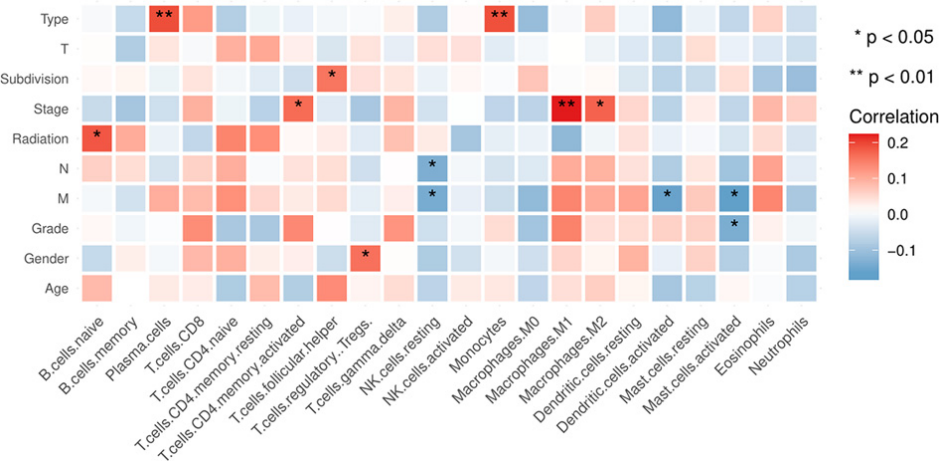
Clinical characteristics of several LM22 immune cells in GC

In terms of clinical features, several LM22 immune cells were associated with clinical characteristics (Figure 5). Pathological stage was associated with M1 macrophages and eosinophils. As the TNM stage increased, the degree of M1 macrophages also enhanced except for Stage IV. The eosinophils were mainly enriched in the Stage IV (Supplementary Figure S2A,B). Follicular helper T cells were activated at the late stage (G3/G4) (Supplementary Figure S2C). Monocytes were associated with radiation therapy. The fraction of total B cells was higher in the samples without radiation therapy than in the samples with radiation therapy, whereas the fraction of total macrophages was higher in the latter than in the former (Supplementary Figure S3, Table 2).

**Table 2:** Comparison of immune cell fractions between with radiation and without radiation in GC

Immune cell type	The fraction in % of all infiltrating immune cells			
	*P*-value	Normal means	Tumor means	logFC
Naive B cells	0.060	0.052	0.073	0.494
Memory B cells	0.674	0.026	0.036	0.441
Plasma cells	0.229	0.055	0.042	−0.403
CD8 T cells	0.590	0.123	0.120	−0.044
Naive CD4 T cells	0.623	0.001	0.000	NA
Resting CD4 memory T cells	0.155	0.166	0.192	0.209
Activated CD4 memory T cells	0.486	0.046	0.032	−0.545
Follicular helper T cells	0.229	0.029	0.023	−0.352
Tregs	0.847	0.063	0.060	−0.062
γδ T cells	0.417	0.001	0.002	1.159
Resting NK cells	0.505	0.012	0.014	0.220
Activated NK cells	0.697	0.022	0.018	−0.300
Monocytes	0.005	0.003	0.009	1.759
M0 macrophages	0.978	0.126	0.118	−0.101
M1 macrophages	0.568	0.071	0.063	−0.183
M2 macrophages	0.601	0.097	0.093	−0.065
Resting dendritic cells	0.447	0.018	0.020	0.132
Activated dendritic cells	0.152	0.019	0.009	−1.116
Resting mast cells	0.579	0.032	0.036	0.153
Activated mast cells	0.796	0.025	0.024	−0.078
Eosinophils	0.918	0.002	0.001	−0.624
Neutrophils	0.770	0.009	0.017	0.891

### Identification of prognostic LM22 immune cell subtypes

**Figure 6:**
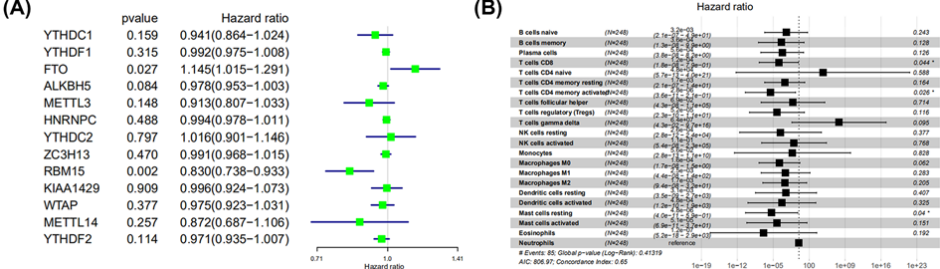
The prognostic associations of subsets of immune cells in univariate Cox regresion and multivariate Cox regression **A**) Univariate Cox regression and (**B**) Multivariate Cox regression.

Univariate Cox regression was performed to identify the prognostic LM22 immune cell subsets in all the tumor samples, and the results showed that two immune cell subsets (γδ T cells and neutrophils) were significantly correlated with OS (*P*<0.05) (Figure 6A, Table 3). In multivariate Cox regression, activated CD4 memory T cells, resting mast cells and CD8 T cells were significantly correlated with OS (Figure 6B, Table 3).

**Table 3:** The prognostic associations of subsets of immune cells in univariate and multivariate Cox regression

The prognostic associations of subsets of immune cells in univariate Cox regression					
Gene	HR	z	*P*-value	Lower	Upper
γδ T cells	43477699	2.226	0.026	8.154	2.32E+14
Neutrophils	526.096	2.059	0.039	1.352	204721
Resting CD4 memory T cells	11.844	1.955	0.051	0.994	141.090
CD8 T cells	0.079	−1.890	0.059	0.006	1.099
Activated CD4 memory T cells	0.017	−1.794	0.073	0.000	1.456
Monocytes	600712.	1.519	0.129	0.021	1.72E+13
Naive B cells	13.344	1.306	0.191	0.274	650.914
Naive CD4 T cells	30726951	0.978	0.328	3.03E-08	3.12E+22
M2 macrophages	4.998	0.928	0.353	0.167	149.236
Tregs	0.113	−0.753	0.452	0.000	33.128
M0 macrophages	0.521	−0.615	0.539	0.065	4.165
Activated dendritic cells	0.117	−0.431	0.666	6.67E-06	2040.
Activated NK cells	7.244	0.426	0.670	0.001	65314
Activated mast cells	2.434	0.424	0.671	0.040	148.105
M1 macrophages	0.346	−0.409	0.683	0.002	56.079
Follicular helper T cells	0.129	−0.409	0.683	6.84E-06	2416
Resting dendritic cells	3.075	0.248	0.804	4.30E-04	22001
Resting NK cells	0.303	−0.220	0.826	7.37E-06	12490
Plasma cells	0.770	−0.172	0.863	0.039	15.235
Eosinophils	0.330	−0.120	0.905	4.43E-09	24566051
Resting mast cells	1.269	0.080	0.936	0.004	432.197
Memory B cells	1.007	0.003	0.997	0.015	65.694

The prognostic associations of subsets of immune cells in multivariate Cox regression					
Immune cells	COEF	HR	HR .95L	HR .95H	*P*-value
Naive B cells	−5.733	0.003	2.15E-07	48.874	0.243
Memory B cells	−7.924	0.000	1.33E-08	9.875	0.128
Plasma cells	−7.491	0.001	3.82E-08	8.152	0.126
CD8 T cells	−9.032	0.000	1.82E-08	0.787	0.044
Naive CD4 T cells	10.776	47852	5.68E-13	4.03E+21	0.588
Resting CD4 memory T cells	−6.388	0.002	2.09E-07	13.506	0.164
Activated CD4 memory T cells	−12.797	0.000	3.58E-11	0.214	0.026
Follicular helper T cells	−2.673	0.069	4.34E-08	109916.931	0.714
Tregs	−9.872	0.000	2.34E-10	11.350	0.116
γδ T cells	17.979	64306589	0.043	9.73E+16	0.095
Resting NK cells	−8.258	0.000	2.81E-12	23880	0.377
Activated NK cells	−2.187	0.112	5.39E-08	233695	0.768
Monocytes	−2.879	0.056	2.77E-13	1.14E+10	0.828
M0 macrophages	−8.731	0.000	1.69E-08	1.543	0.062
M1 macrophages	−5.998	0.002	4.391E-08	140.539	0.283
M2 macrophages	−6.357	0.002	9.36E-08	32.178	0.205
Resting dendritic cells	−5.789	0.003	3.48E-09	2695	0.407
Activated dendritic cells	−7.651	0.000	1.16E-10	1948	0.325
Resting mast cells	−12.238	0.000	3.99E-11	0.588	0.040
Activated mast cells	−9.893	0.000	6.94E-11	36.799	0.151
Eosinophils	−15.905	0.000	5.24E-18	2923	0.192
Neutrophils	NA	NA	NA	NA	NA

The ROC curves were used to evaluate the prognostic power of prognostic LM22 immune cell subsets. The AUC of prognostic LM22 immune cell subset biomarker model is shown in Supplementary Figure S4. In GC, resting mast cells, naïve B cells, monocytes, neutrophils, activated CD4 memory T cellsnd follicular helper T cells had an AUC of more than 0.550. Monocytes had the highest performance in the risk prediction of patients with GC.

### Immune cell infiltration patterns in molecular GC subtypes

**Figure 7:**
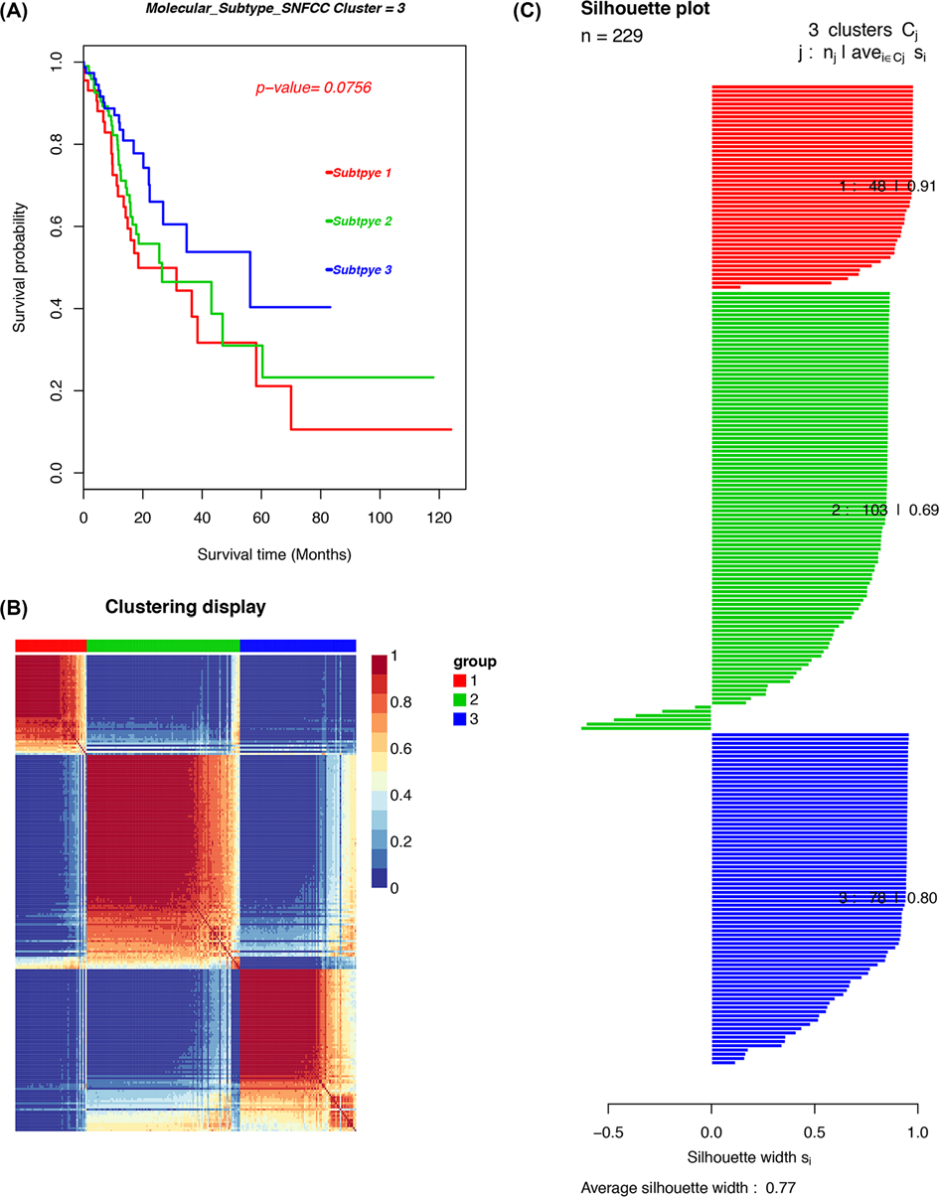
The cancer subtypes using SNFCC+ algorithm (**A**) Log-rank test *P*-value for Kaplan–Meier survival analysis. (**B**) Clustering heatmap visualizing the degree of the partitioning of the sample clusters. (**C**) Average silhouette width representing the coherence of clusters.

The molecular classification of GC was identified by performing an unsupervised consensus clustering of 221 tumor samples to explore the infiltration of different immune cell populations in a TME in GC. The optimal number of clusters was determined with *K*. After the relative altercations in the area under the cumulative distribution function (CDF) curve and the consensus heatmap were evaluated, a three-cluster solution (*K* = 3) was selected, but it did not remarkably increase in the area under the CDF curve (Supplementary Figure S5). This finding classified 48 patients (21%) in cluster I, 103 patients (45%) in cluster II and 78 patients (34%) in cluster III for the GC cohort. The consensus matrix heatmap revealed cluster I, II and III with individualized clusters. The sample of each cluster is shown in Figure 7. The clusters were associated with distinct survival patterns. The patients classified under cluster II had a good prognosis compared with those in clusters I and III.

### Differentially expressed analysis of genes/LM22 immune cell fractions in each GC subgroup

**Figure 8:**
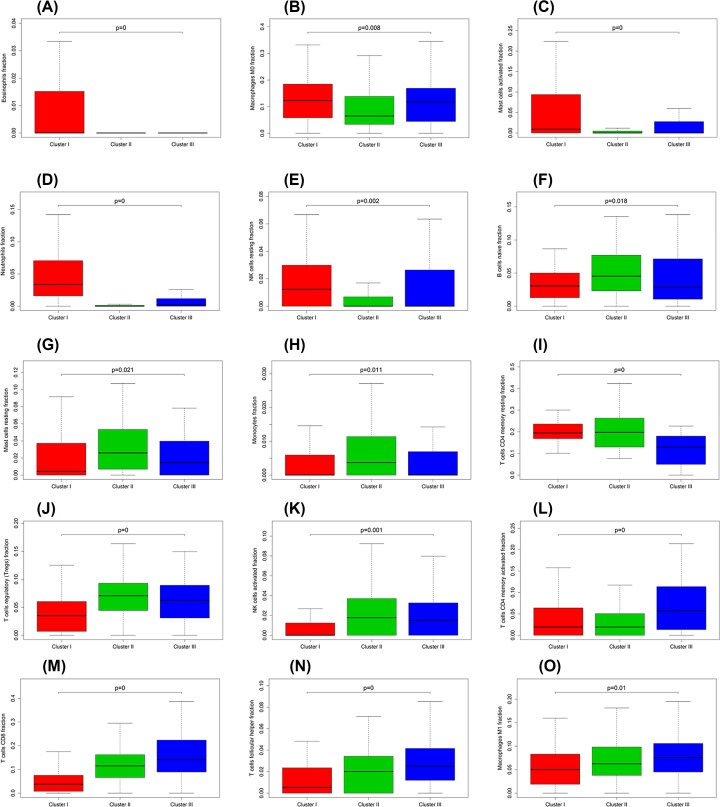
Box plot depicting the association between immune cell subsets and three clusters, depicted *P*-values are from the Kruskal–Wallis tests (**A**–**O**) are for eosinophils, M0 macrophages, activated mast cells, neutrophils, resting NK cells, naïve B cells, resting mast cells, monocytes, resting CD4 memory T cells, Tregs, activated NK cells, activated CD4 memory T cells, CD8 T cells, follicular helper T cells and M1 macrophages, respectively.

The Kruskal–Wallis test was conducted to identify the quantitative genes/LM22 immune cells significantly associated with each subclass. For differential LM22 immune cell in GC, cluster I was defined with a high level of eosinophils, M0 macrophages, activated mast cells, neutrophils and resting NK cells. Cluster II was enriched with naïve B cells, resting mast cells, monocytes, resting CD4 memory T cells, Tregs and activated NK cells. Cluster III was defined with a high level of activated CD4 memory T cells, CD8 T cells, follicular helper T cells and M1 macrophages (Figure 8). The heatmap is also illustrated in Supplementary Figure S6.

**Figure 9:**
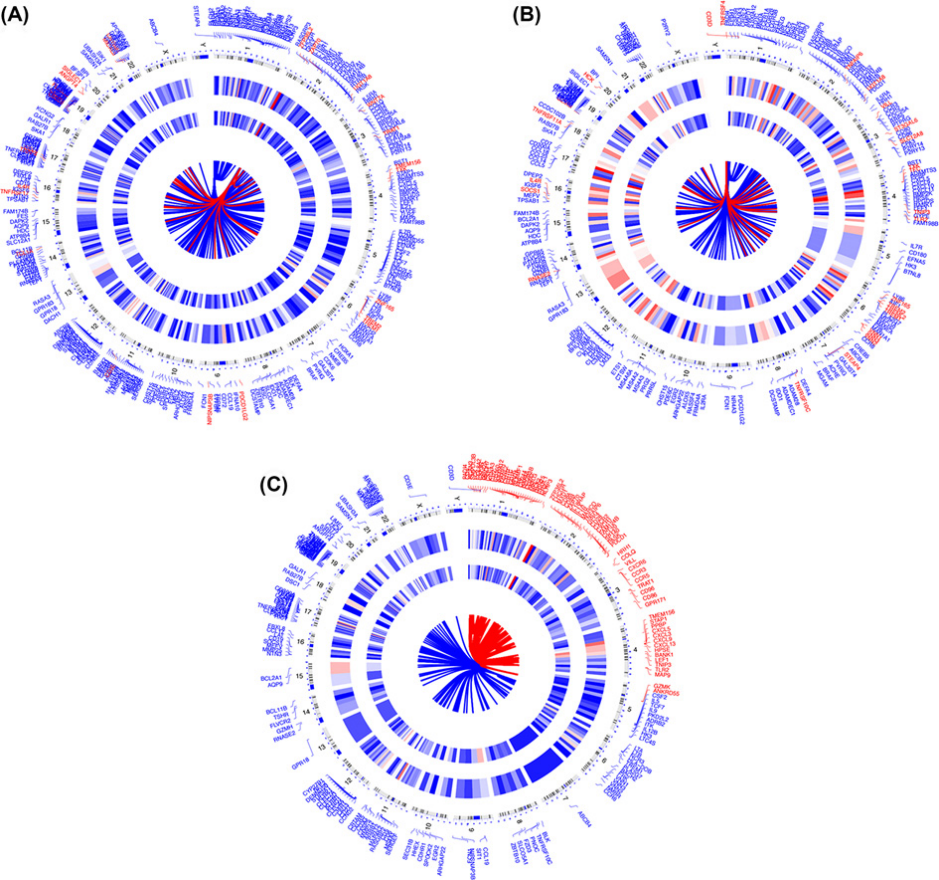
DEGs in concentric circles radiating among three GC subgroups (**A**–**C**) are for subgroup I vs subgroups II, subgroup 1 vs subgroups III, subgroup II vs subgroups III.

An unpaired Student’s *t* test was conducted to identify the quantitative genes significantly associated with each subtype and examine the molecular differences between GC molecular subtypes and derived subtype-specific biomarkers. The unmatched subgroups were subjected to DEG analysis with a threshold of absolute log-fold change cut-off >0.1 and false discovery rate (FDR) = 0.05. Figure 9 shows DEGs in concentric circles radiating among the three clusters. A total of 158 mRNAs (192 up-regulated and 77 down-regulated genes) in subgroup I were differentially expressed compared with those in subgroups Ⅱ. In subgroup I compared with subgroups III, 216 differentially expressed mRNAs (28 up-regulated and 187 down-regulated genes) were detected. In subgroup Ⅱ compared with subgroup III, 313 differentially expressed mRNAs (26 up-regulated and 287 down-regulated genes) were observed.

### GO, KEGG and GSVA of DEGs for molecular subtypes identification

**Figure 10:**
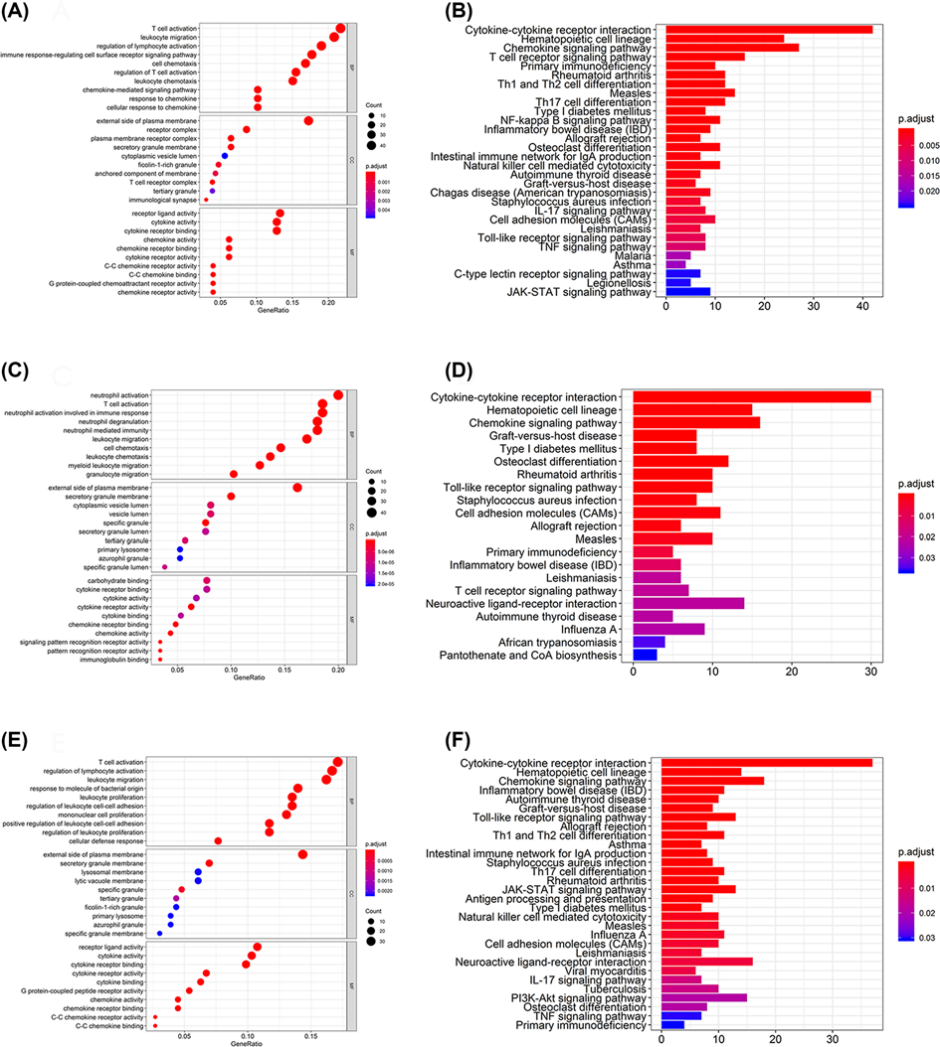
The GO and KEGG analysis for three GC clusters (**A,B**) Are for cluster I vs cluster II, (**C,D**) are for cluster I vs cluster III and (**E,F**) are for cluster II vs cluster III.

A total of 639 GO terms of biological processes, 17 GO terms of cellular components and 54 GO terms of molecular functions in subgroup I were significantly compared with those in subgroup Ⅱ (adjusted *P*<0.05). In subgroup I compared with subgroup III, 526 GO terms of biological processes, 31 GO terms of cellular components and 37 GO terms of molecular functions were significant. In subgroup Ⅱ compared with subgroup III, 605 GO terms of biological processes, 14 GO terms of cellular components and 31 GO terms of molecular functions were significant. The top GO terms included cytokine activity, immune/inflammatory response and chemokine activity. All the pathways obtained through KEGG analysis were associated with immune responses (Figure 10). The unpaired Student’s *t* test was conducted to identify quantitative genes and examine the molecular differences between GC subtypes and derived subtype-specific biomarkers.

**Figure 11:**
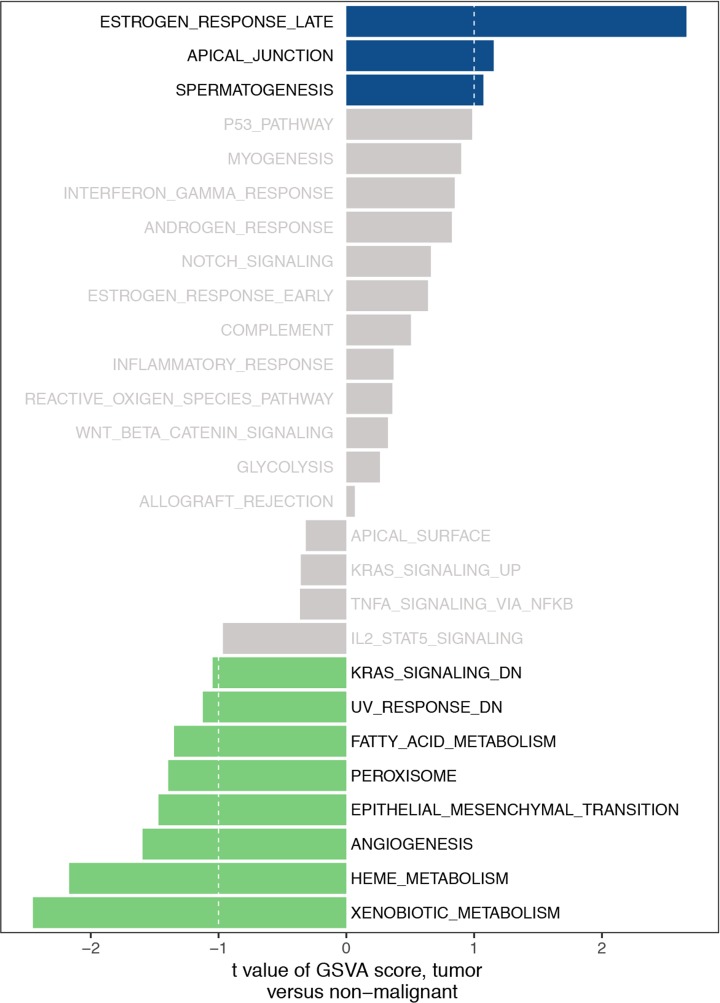
The GSVA analysis for three GC clusters

Three clusters were subjected to GSVA by using the GSVA package of R software. The number of enriched pathways progressively increased from subtype I to subtype III. The most significantly enriched gene sets were ordered on the basis of significance (*P* and adjusted *P*-values of FDR) and listed in Table 4. In Figure 11, several hallmark gene sets, including estrogen response late, apical junction, epithelial–mesenchymal transition (EMT), KRAS signaling and angiogenesis were observed.

**Table 4:** The GSVA analysis for three GC clusters

	logFC	AveExpr	*t*	*P*-value	adj. *P*-value	B
HALLMARK_ESTROGEN_RESPONSE_LATE	0.421	−0.008	2.661	0.010	0.232	−2.710
HALLMARK_XENOBIOTIC_METABOLISM	−0.378	0.019	−2.452	0.017	0.232	−3.093
HALLMARK_HEME_METABOLISM	−0.228	−0.004	−2.169	0.034	0.307	−3.573
HALLMARK_ANGIOGENESIS	−0.234	0.043	−1.595	0.116	0.696	−4.392
HALLMARK_EPITHELIAL_MESENCHYMAL_TRANSITION	−0.231	0.032	−1.470	0.147	0.696	−4.541
HALLMARK_PEROXISOME	−0.211	0.018	−1.394	0.169	0.696	−4.626
HALLMARK_FATTY_ACID_METABOLISM	−0.221	0.008	−1.348	0.183	0.696	−4.676
HALLMARK_APICAL_JUNCTION	0.161	−0.022	1.153	0.253	0.696	−4.868
HALLMARK_UV_RESPONSE_DN	−0.191	0.022	−1.123	0.266	0.696	−4.895
HALLMARK_SPERMATOGENESIS	0.132	0.001	1.072	0.288	0.696	−4.939
HALLMARK_KRAS_SIGNALING_DN	−0.102	0.020	−1.046	0.300	0.696	−4.961
HALLMARK_P53_PATHWAY	0.141	−0.020	0.985	0.329	0.696	−5.011
HALLMARK_IL2_STAT5_SIGNALING	−0.101	0.013	−0.966	0.338	0.696	−5.026
HALLMARK_MYOGENESIS	0.158	−0.005	0.899	0.372	0.696	−5.076
HALLMARK_INTERFERON_GAMMA_RESPONSE	0.104	−0.019	0.848	0.400	0.696	−5.111
HALLMARK_ANDROGEN_RESPONSE	0.116	−0.018	0.825	0.412	0.696	−5.126
HALLMARK_NOTCH_SIGNALING	0.108	−0.021	0.661	0.511	0.788	−5.225
HALLMARK_ESTROGEN_RESPONSE_EARLY	0.113	0.029	0.639	0.525	0.788	−5.236
HALLMARK_COMPLEMENT	0.052	0.005	0.505	0.615	0.813	−5.298
HALLMARK_INFLAMMATORY_RESPONSE	0.031	−0.007	0.370	0.713	0.813	−5.346
HALLMARK_TNFA_SIGNALING_VIA_NFKB	−0.042	−0.031	−0.362	0.718	0.813	−5.349
HALLMARK_REACTIVE_OXIGEN_SPECIES_PATHWAY	0.050	−0.003	0.360	0.720	0.813	−5.349
HALLMARK_KRAS_SIGNALING_UP	−0.039	0.014	−0.356	0.723	0.813	−5.350
HALLMARK_WNT_BETA_CATENIN_SIGNALING	0.055	−0.016	0.325	0.746	0.813	−5.359
HALLMARK_APICAL_SURFACE	−0.047	0.003	−0.317	0.752	0.813	−5.361
HALLMARK_GLYCOLYSIS	0.043	−0.019	0.263	0.794	0.824	−5.374
HALLMARK_ALLOGRAFT_REJECTION	0.008	0.002	0.066	0.947	0.947	−5.400

## Discussion

The majority of patients with GC have been actively treated using a multimodality strategy [20], but their 5-year survival rates have not increased [21]. Although new immunotherapies have provided a new basis for treating patients with GC, their potential mechanism is still unclear, and further studies should be performed to develop the corresponding targeted therapy. Immune cells, which have multiple types and different functions, are the main TME components and major host cells recruited and activated by a TME. The immune system can promote and inhibit tumor growth, so this system is important for prognosis. Studies have suggested that TIICs have great potential for application in clinical outcome and therapeutic response prediction among individual patients. Jiang et al. [22] found that the density and distribution of TIICs may be useful in predicting the survival of patients with GC. Previous studies revealed that TIICs, such as mature T cells, dendritic cells and memory T cells with increased infiltration, are associated with good prognosis, whereas immunosuppressive Tregs are opposite [23,24]. Previous studies also applied immunohistochemical methods to evaluate TIICs and identify the TIIC subgroup with a single surface marker because of technical limitations. However, these methods are poorly effective in identifying closely related cell types. As such, inconsistent results have been presented in clinical studies.

A combination of CIBERSOFT algorithms can overcome the shortcomings of traditional immunohistochemical methods and accurately address the relative proportions of different TIICs [12,25]. Therefore, in the current study, CIBERSORT was used to infer the proportion of 22 immune cell subsets from the GC transcriptome. To our knowledge, this study was the first to comprehensively analyze the clinical effect of immune responses on GC.

In our study, the CIBERSOFT method was conducted to evaluate the infiltration of different immune cells in paired GC and adjacent normal tissues, and the results showed that the scores of immune cells significantly differed within and between groups. Our work also revealed the details of the infiltration of various subsets of LM22 immune cells in GC. The profiles of immune infiltration in the TCGA GC cohort varied significantly between 22 paired GC tissues. Plasma cells and resting CD4 memory T cells increased in the paired paracancerous tissues, whereas activated CD4 memory T cells and M0 macrophages decreased in the GC samples. In terms of clinical characteristics, the correlation between 22 immune cell subtypes and clinical characteristics was also analyzed. M1 macrophages and eosinophils were related to TNM stage. Follicular helper T cells were activated at the late stage (G3/G4), and monocytes were associated with radiotherapy. Among LM22 immune cells, Tregs were significantly associated with the OS of patients with GC. Therefore, these results demonstrated that aberrant immune infiltration and heterogeneity in GC as a tightly regulated process might play important roles in tumor development and this process had clinical importance.

Recent studies have found that the interaction of cytokines showed an important role in the development and progression of malignant tumors. Previous studies have revealed that IL-17 was involved in tumor occurrence, metastasis, angiogenesis, immune-resistance and other processes [26–28], and confirmed that IL-17 signaling pathway also played an important role in the occurrence and progression of GC [29], breast cancer [30] and pancreatic cancer [31]. Meanwhile, Liu et al. [32] obtained the data of *Helicobacter pylori* GC samples from TCGA and revealed that cytokine–cytokine receptor interaction was enriched in *H. pylori* (+) GC through GO and KEGG analysis. Wu et al. [33] used Human gene chip Affymetrix HTA 2.0, obtained 1312 DEGs in GES-1 cell lines with *H. pylori* and TMAO co-treatment compared with the control, and Toll-like receptor signaling pathway was showed to be the most important biological processes. Yu et al. [34] used multimarker analysis of genomic annotation to analyze pathways, and identified that chemokine signaling pathway was associated with GC risk. In our study, DEGs were conducted through GO and KEGG analysis. The top GO terms included cytokine activity, immune/inflammatory response and chemokine activity. All the pathways obtained through KEGG analysis were associated with immune responses. And we also found that cytokine–cytokine receptor interaction, the Toll-like receptor signaling pathway, IL-17 signaling pathway and chemokine signaling pathway were significantly enriched, which was predicted that these signaling pathways might play an important role in future immunotherapy research.

In summary, the CIBERSORT algorithm was used to analyze the LM22 immune cell subsets in GC and provided information about the immune cell landscape of GC. Our findings also revealed the important correlation of clinical outcome with the LM22 immune cell infiltration model in a TME. The present study helped clarify the tumor responses to immunotherapy and might be used as a basis for discovering highly possible targets of new drugs.

## Supplementary Material

Supplementary Figures S1-S6

## Data Availability

The data used to perform the analyses described herein are publically available from official TCGA data portal.

## Abbreviations

AUC: area under the curve
CDF: cumulative distribution function
CIBERSORT: Cell type Identification By Estimating Relative Subsets Of known RNA Transcripts
DEG: differentially expressed gene
FDR: false discovery rate
GC: gastric cancer
GO: Gene Ontology
GSVA: gene set variation analysis
IL: interleukin
KEGG: Kyoto Encyclopedia of Genes and Genomes
KRAS: Kirsten rat sarcoma viral oncogene homolog
OS: overall survival
TCGA: The Cancer Genome Atlas
TIIC: tumor-infiltrating immune cell
TMAO: trimethylamine-N-oxide
TME: tumor microenvironment
TNM: tumor node metastasis
Treg: regulatory T cell
