# Supplementary material for: Genomic analysis and clinical implications of immune cell infiltration in gastric cancer
Source: Biosci Rep. 2020 May 20;40(5):BSR20193308. doi: 10.1042/BSR20193308 (PMC7240200; doi:10.1042/BSR20193308)
Supplement: Supplementary Figures S1-S6 [file BSR-2019-3308_supp.pdf]

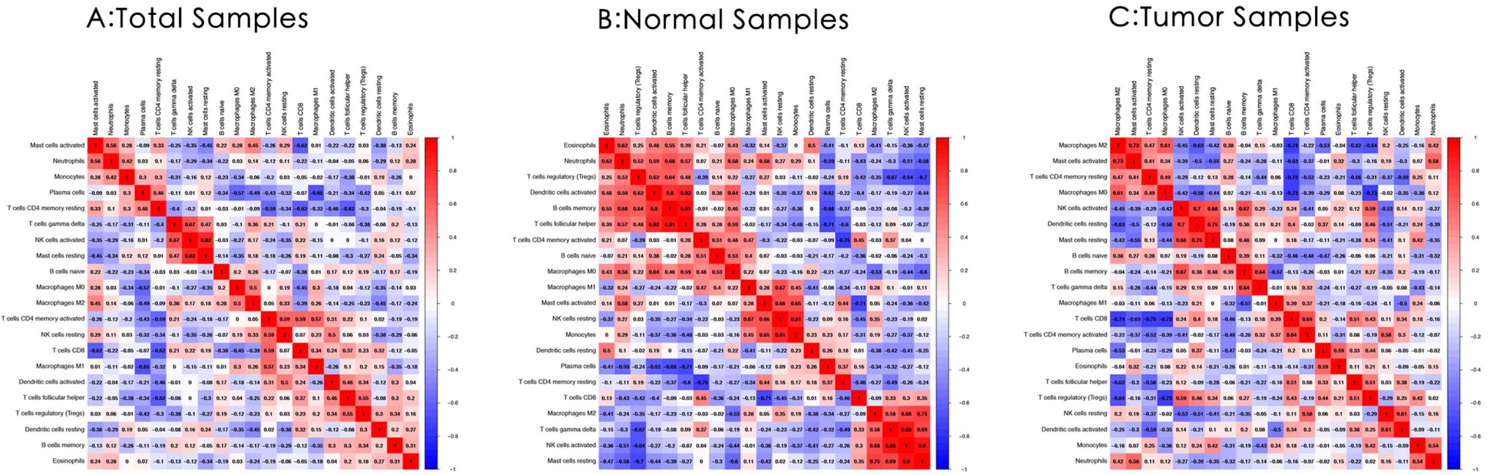
A:Total Samples
B:Normal Samples
C:Tumor Samples
Mast cells activated
Neutrophils
Monocytes
Plasma cells
T cells CD4 memory resting
T cells gamma delta
NK cells activated
Mast cells resting
B cells naive
Macrophages M0
Macrophages M2
T cells CD4 memory activated
NK cells resting
T cells CD8
Macrophages M1
Dendritic cells activated
T cells follicular helper
T cells regulatory (Tregs)
Dendritic cells resting
B cells memory
Eosinophils
1
0.8
0.6
0.4
0.2
0
-0.2
-0.4
-0.6
-0.8
-1

Supplementary Figure 1 Correlation matrix of all 22 immune proportions and immune cytolytic activity in the TCGA GC cohort, including total samples (**A**), normal samples (**B**) and tumor samples (**C**).

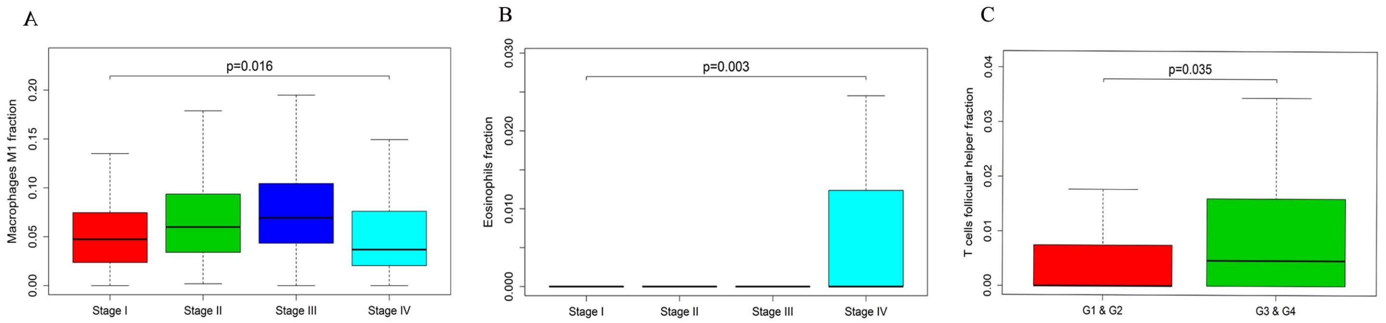
A
p=0.016
Macrophages M1 fraction
0.00
0.05
0.10
0.15
0.20
Stage I
Stage II
Stage III
Stage IV
B
p=0.003
Eosinophils fraction
0.000
0.010
0.020
0.030
Stage I
Stage II
Stage III
Stage IV
C
p=0.035
T cells follicular helper fraction
0.00
0.01
0.02
0.03
0.04
G1 & G2
G3 & G4

Supplementary Figure 2 Box plot depicting the correlation between several LM22 immune cells and GC classification based the distribution of CIBERSORT *P*-value. **A-B**. M1 macrophages and eosinophils in the pathological stage. **C.** follicular helper T cells at the late stage (G3/G4).

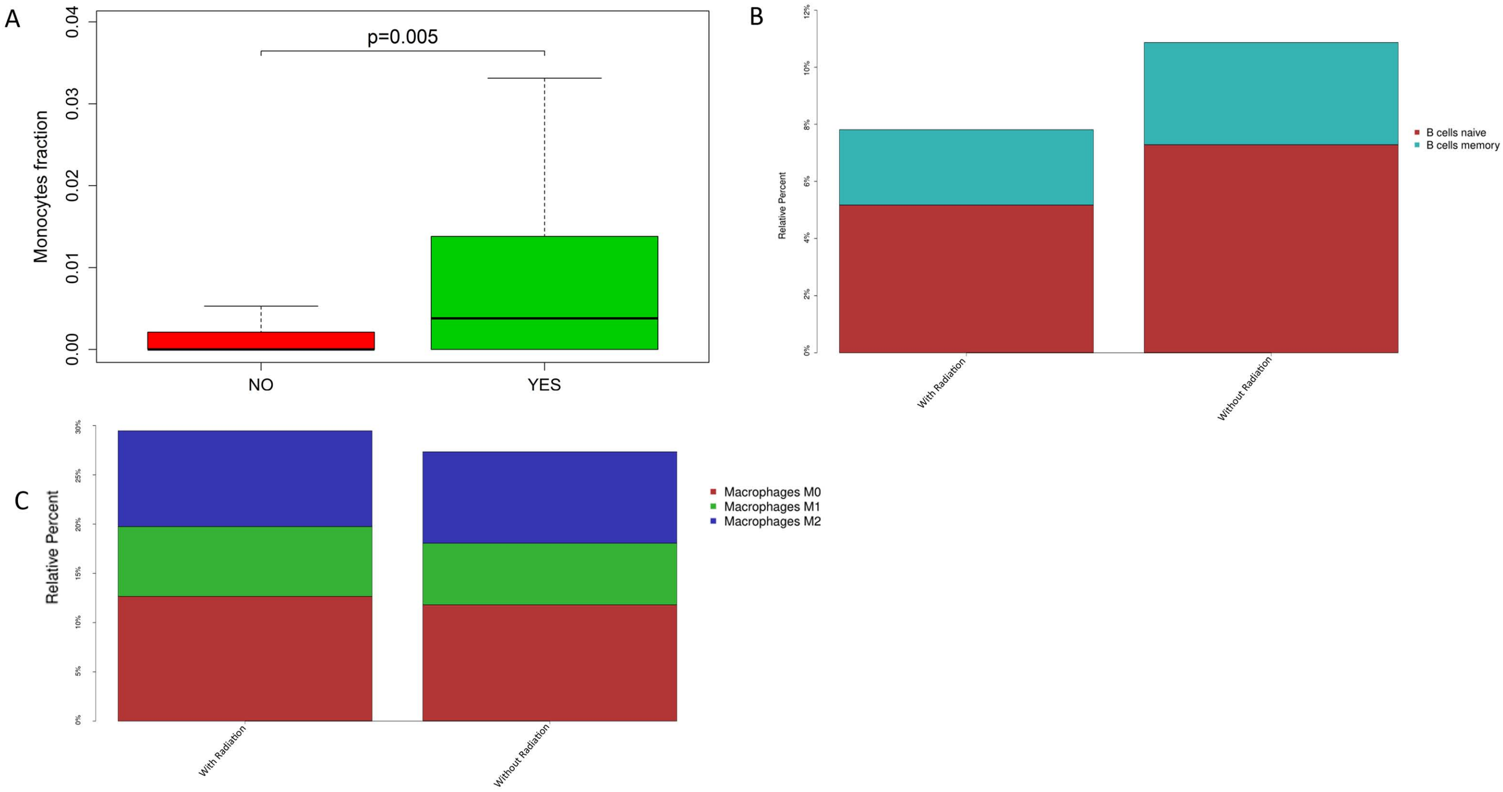
A
p=0.005
Monocytes fraction
0.00
0.01
0.02
0.03
0.04
NO
YES
B
Relative Percent
0%
2%
4%
6%
8%
10%
12%
B cells naive
B cells memory
With Radiation
Without Radiation
C
Relative Percent
0%
5%
10%
15%
20%
25%
30%
Macrophages M0
Macrophages M1
Macrophages M2
With Radiation
Without Radiation

Supplementary Figure 3 The difference of immune infiltration between GC with radiation therapy and without radiation therapy samples. **A**. Box plot of the distribution of CIBERSORT *P* value for monocytes. **B-C**. The total difference in total B cells and total macrophages, respectively.

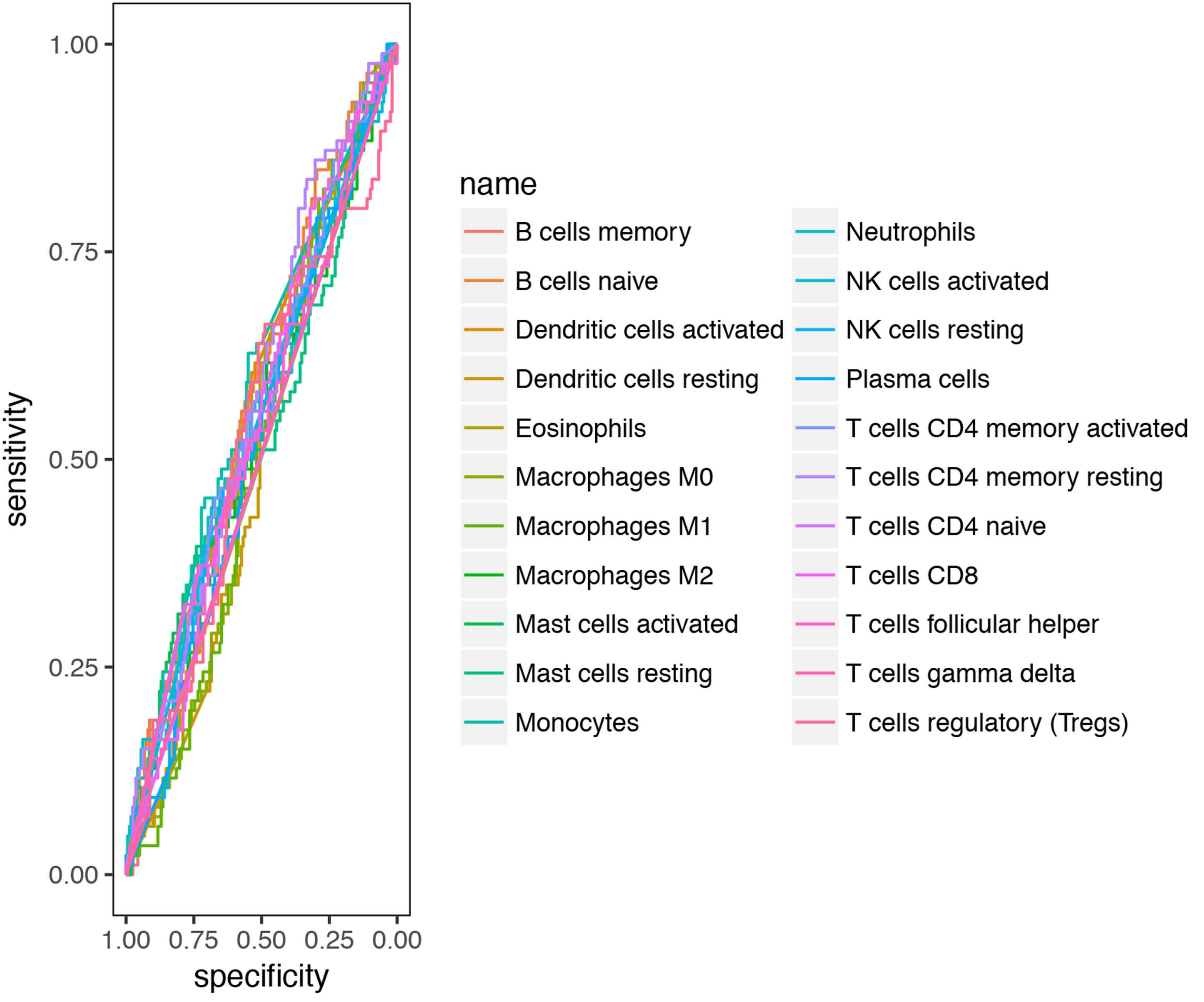

sensitivity
1.00
0.75
0.50
0.25
0.00
specificity
1.00
0.75
0.50
0.25
0.00
name
B cells memory
B cells naive
Dendritic cells activated
Dendritic cells resting
Eosinophils
Macrophages M0
Macrophages M1
Macrophages M2
Mast cells activated
Mast cells resting
Monocytes
Neutrophils
NK cells activated
NK cells resting
Plasma cells
T cells CD4 memory activated
T cells CD4 memory resting
T cells CD4 naive
T cells CD8
T cells follicular helper
T cells gamma delta
T cells regulatory (Tregs)

Supplementary Figure 4 AUC curves of biomarkers prognostic model built by LM22 immune cell subsets.

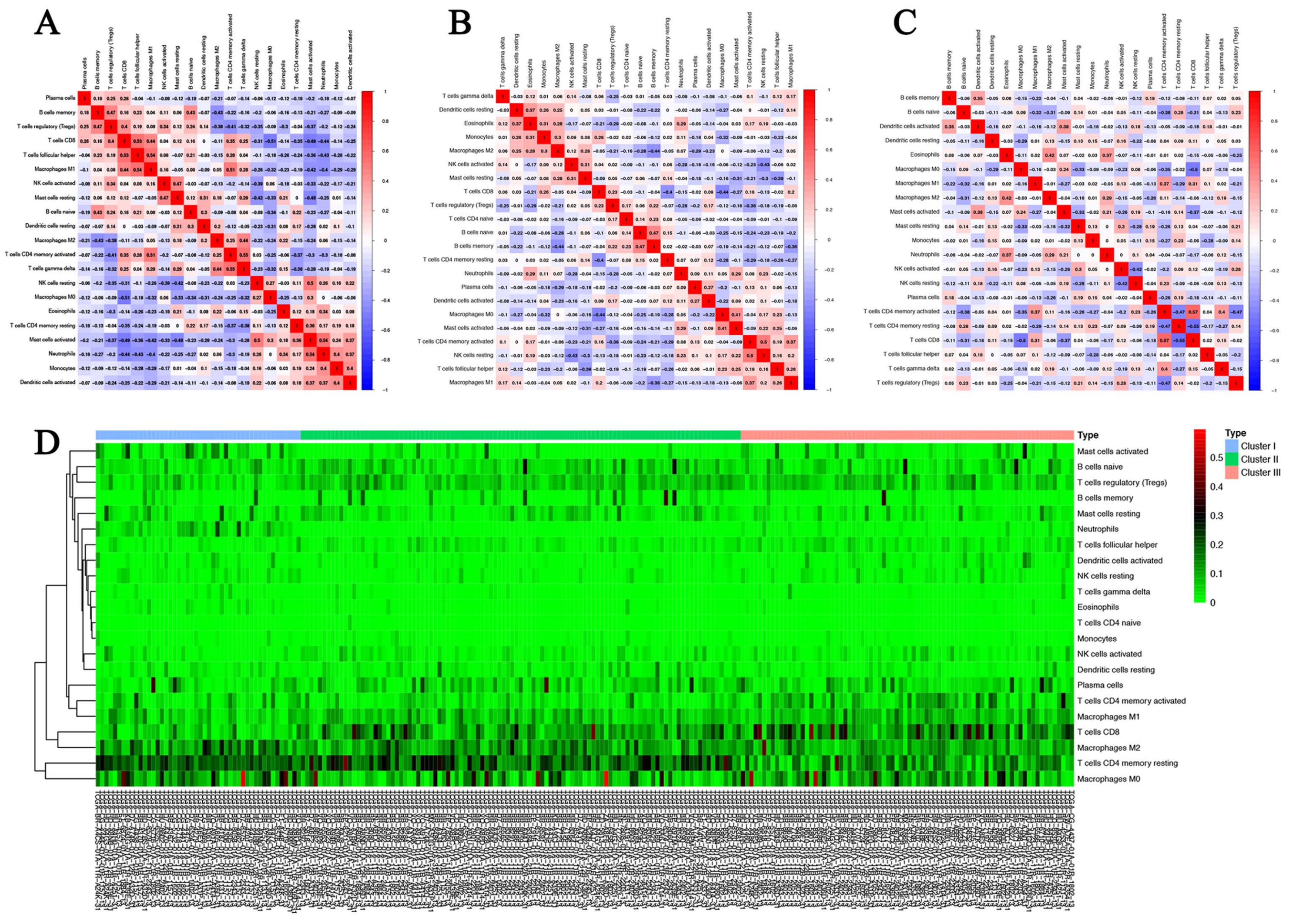
A
Plasma cells
B cells memory
T cells regulatory (Tregs)
T cells CD8
T cells follicular helper
Macrophages M1
NK cells activated
Mast cells resting
B cells naive
Dendritic cells resting
Macrophages M2
T cells CD4 memory activated
T cells gamma delta
NK cells resting
Macrophages M0
Eosinophils
T cells CD4 memory resting
Mast cells activated
Neutrophils
Monocytes
Dendritic cells activated
B
T cells gamma delta
Dendritic cells resting
Eosinophils
Monocytes
Macrophages M2
NK cells activated
Mast cells resting
T cells CD8
T cells regulatory (Tregs)
T cells CD4 naive
B cells naive
B cells memory
T cells CD4 memory resting
Neutrophils
Plasma cells
Dendritic cells activated
Macrophages M0
Mast cells activated
T cells CD4 memory activated
NK cells resting
T cells follicular helper
Macrophages M1
C
B cells memory
B cells naive
Dendritic cells activated
Dendritic cells resting
Eosinophils
Macrophages M0
Macrophages M1
Macrophages M2
Mast cells activated
Mast cells resting
Monocytes
Neutrophils
NK cells activated
NK cells resting
Plasma cells
T cells CD4 memory activated
T cells CD4 memory resting
T cells CD8
T cells follicular helper
T cells gamma delta
T cells regulatory (Tregs)
D
Type
Mast cells activated
B cells naive
T cells regulatory (Tregs)
B cells memory
Mast cells resting
Neutrophils
T cells follicular helper
Dendritic cells activated
NK cells resting
T cells gamma delta
Eosinophils
T cells CD4 naive
Monocytes
NK cells activated
Dendritic cells resting
Plasma cells
T cells CD4 memory activated
Macrophages M1
T cells CD8
Macrophages M2
T cells CD4 memory resting
Macrophages M0
0.5
0.4
0.3
0.2
0.1
0
Type
Cluster I
Cluster II
Cluster III

Supplementary Figure 5 The cluster counts evaluated. (**A**) consensus heatmap (**B**) CDF curve of *K*=2-5. (**C**) The relative change in area under the CDF curve of *K*= 2-5.

A

B

C

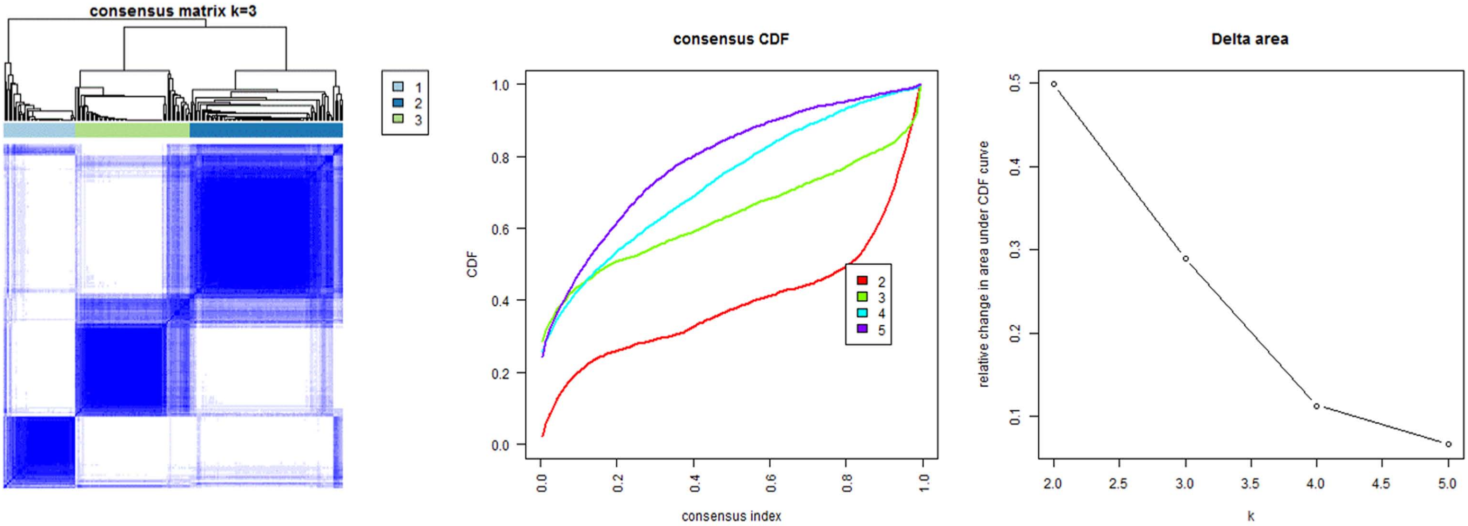

consensus matrix k=3
1
2
3
consensus CDF
CDF
1.0
0.8
0.6
0.4
0.2
0.0
consensus index
2
3
4
5
Delta area
relative change in area under CDF curve
0.5
0.4
0.3
0.2
0.1
2.0
2.5
3.0
3.5
4.0
4.5
5.0
k

Supplementary Figure 6 Heatmap of 22 immune cells and clusters. **A-C** is for the correlation matrix of the 22 immune cells proportions in cluster I - III, respectively. **D**. Heatmap of the 22 immune cells proportions.
